# GENDER AND AGE EFFECTS ON PTG EXPLAINED BY PSYCHOLOGICAL FACTORS IN CARDIAC PATIENTS

**DOI:** 10.1093/geroni/igac059.3046

**Published:** 2022-12-20

**Authors:** Amy Ai, Crim Sabuncu

**Affiliations:** Florida State University, Tallahassee, Florida, United States; Florida State University, Tallahassee, Florida, United States

## Abstract

The present study explored the role of gender on posttraumatic growth (PTG) 30-months after cardiac surgery and mediation of preoperative psychosocial factors. Two previous meta-analyses found that woman tended to have more growth than men, and that PTG was associated with improved overall health, but most studies were cross-sectional and small-scale. Using a prospective design, we followed 262 patients undergoing cardiovascular operations with surveys and objectively measured medical indices from the Society of Thoracic Surgeons’ (STS) national database. Participants completed a follow-up survey on PTG 30-month after surgery. Bivariate correlations related PTG with female gender, age, minority race, marriage, and faith-based factors, but no medical or other psychosocial factors. In hierarchical regressions, gender and age were linked with PTG, alongside other demographics in Step-1. These effects sustained after entry of STS indices, in Step-2, but the gender role diminished after adding medical comorbidities, preoperative depression, optimism, hope, and social support in Step-3. No other factor in these steps had an impact. The age effect vanished when faith factors were entered, but only positive spiritual coping (PSC) was related to PTG. Finally, the role of PSC on PTG was mediated by perceived spiritual support. The final model was significant, accounting for one-third of the variance [F (20, N = 215) = 4.896, p < .001, R2 = .334] in PTG. This suggests that more research should aim to explore the effect of gender and age on PTG in general. For cardiac patients, care providers may encourage positive faith-based coping prior to cardiovascular surgery.

